# Retraction Note: Swainsonine represses glioma cell proliferation, migration and invasion by reduction of miR-92a expression

**DOI:** 10.1186/s12885-024-12034-x

**Published:** 2024-02-29

**Authors:** Libo Sun, Xingyi Jin, Lijuan Xie, Guangjun Xu, Yunxia Cui, Zhuo Chen

**Affiliations:** 1 Department of Neurosurgery, Union Hospital of Jilin University, No.126, Xiantai Street, 130033 Changchun, Jilin Province China; 2 Department of Vascular Surgery, Union Hospital of Jilin University, 130033 Changchun, Jilin Province China; 3 Department of Science and Education, Union Hospital of Jilin University, 130033 Changchun, Jilin Province China

**Retraction Note**: ***BMC Cancer*** **19, 247 (2019)**

10.1186/s12885-019-5425-7.

The Editors have retracted this article following an investigation report by the Ministry of Science and Technology of the People’s Republic of China. The investigation found attempts to sell and purchase the data presented in this article. Therefore, the Editors no longer have confidence in the integrity of the data in this article.

None of the authors have responded to any correspondence from the editor/publisher about this retraction.

